# Acute Pyelonephritis Complicated With Spontaneous Subcapsular Renal Hematoma: A Case Report

**DOI:** 10.7759/cureus.73825

**Published:** 2024-11-16

**Authors:** Joana Subtil, Rui Carvalho, Ana Filipa Rebelo, Fernando Guimarães

**Affiliations:** 1 Internal Medicine, Centro Hospitalar de Trás-os-Montes e Alto Douro, Vila Real, PRT; 2 Internal Medicine, Centro Hospitalar de Trás-Os-Montes e Alto Douro, Vila Real, PRT

**Keywords:** acute pyelonephritis, complicated urinary tract infection, kidney hemorrhage, klebsiella pneumoniae bacteraemia, spontaneous subcapsular renal hematoma

## Abstract

Spontaneous subcapsular renal hematoma is a rare condition defined as spontaneous hemorrhage confined to the subcapsular and/or perirenal space. Its etiology can vary, ranging from renal tumors to vascular diseases, renal cysts, or infections. Here, we report the case of a 54-year-old male who presented to the emergency department with a two-day history of abdominal pain and fever. He was hemodynamically stable, with no abnormalities on physical examination except for mild pain on deep palpation of the left upper quadrant. Laboratory tests showed leukocytosis (24,200/μL), creatinine 1 mg/dL, C-reactive protein 20.77 mg/dL, and lactate 0.6 mmol/L. Urinalysis revealed leukocytes 10-25/HPF. An abdominal CT showed a hypodense area on the left kidney, suggesting focal pyelonephritis. He was admitted for acute pyelonephritis (APN), and empirical antibiotic therapy with ceftriaxone 2 g once daily was started. During hospitalization, Klebsiella pneumoniae was isolated from both blood and urine cultures, and antibiotic therapy was switched to cefotaxime according to susceptibility testing. Despite undergoing targeted antibiotic therapy, the patient maintained a fever, so another abdominal CT was performed, showing an external subcapsular collection of 9.1 cm × 7.9 cm × 2.8 cm, consistent with a subcapsular hematoma. Despite a drop in hemoglobin from 14 to 10 g/dL, the patient remained hemodynamically stable, so it was decided to continue conservative medical management. Spontaneous subcapsular renal hematoma, though rare in acute pyelonephritis, should be considered if symptoms persist despite antibiotics. Treatment may range from conservative monitoring to surgical intervention, depending on stability and the underlying cause.

## Introduction

Subcapsular renal hematoma usually occurs in traumatic or iatrogenic injury and is marked by the accumulation of blood between the renal capsule and the renal parenchyma. Spontaneous subcapsular hematomas are rare, and the most common etiology is a benign or malignant neoplasm, followed by vascular disease (including polyarteritis nodosa, aneurysm, arteriovenous malformation, portal hypertension, Wegener’s granulomatosis, and infarction).

Acute pyelonephritis (APN) is an infection of the renal parenchyma and collecting system, typically caused by bacterial pathogens. APN can lead to several complications, such as sepsis, renal abscess, pyonephrosis, and emphysematous pyelonephritis, particularly in high-risk patients (diabetes mellitus, anatomic or congenital abnormalities, immunosuppression, and advanced age) [1]. In rare cases, pyelonephritis can lead to the development of subcapsular renal hematoma. In meta-analyses with 165 cases, severe pyelonephritis was identified as a cause of spontaneous subcapsular hematoma in four patients (2.4%), of whom one had the xanthogranulomatous variant and two had abscess [2]. The pathophysiology behind this complication involves the infection causing inflammation and weakening of the renal vasculature, which can lead to spontaneous bleeding and hematoma formation [3]. This condition can present with worsening flank pain, a significant drop in hemoglobin levels, and signs of hemodynamic instability despite appropriate antibiotic therapy [3].

Here, we report a rare case of subcapsular hematoma complicating acute pyelonephritis in a patient without high-risk factors.

## Case presentation

The patient was a 54-year-old male with a history of hypertension, dyslipidemia, and non-nephrotic proteinuria with normal renal function and was being treated with lisinopril 20 mg once daily. He presented to the emergency department with a two-day history of abdominal pain in the left upper quadrant and fever. No trauma was associated.

On physical examination, vital signs were stable: blood pressure 137/71 mmHg, heart rate 91/min, SpO2 (room air) 98%, temperature of 39 °C with shivering, well perfused with capillary refill time <2 seconds. He was alert, oriented, and cooperative. The chest was symmetric with no respiratory effort. Breath sounds were clear to auscultation bilaterally. Heart sounds were regular with normal S1 and S2 and no murmurs. The abdomen was flat, with mild tenderness on the left upper quadrant, without rebound tenderness or guarding. No edema was present.

**Figure 1:**
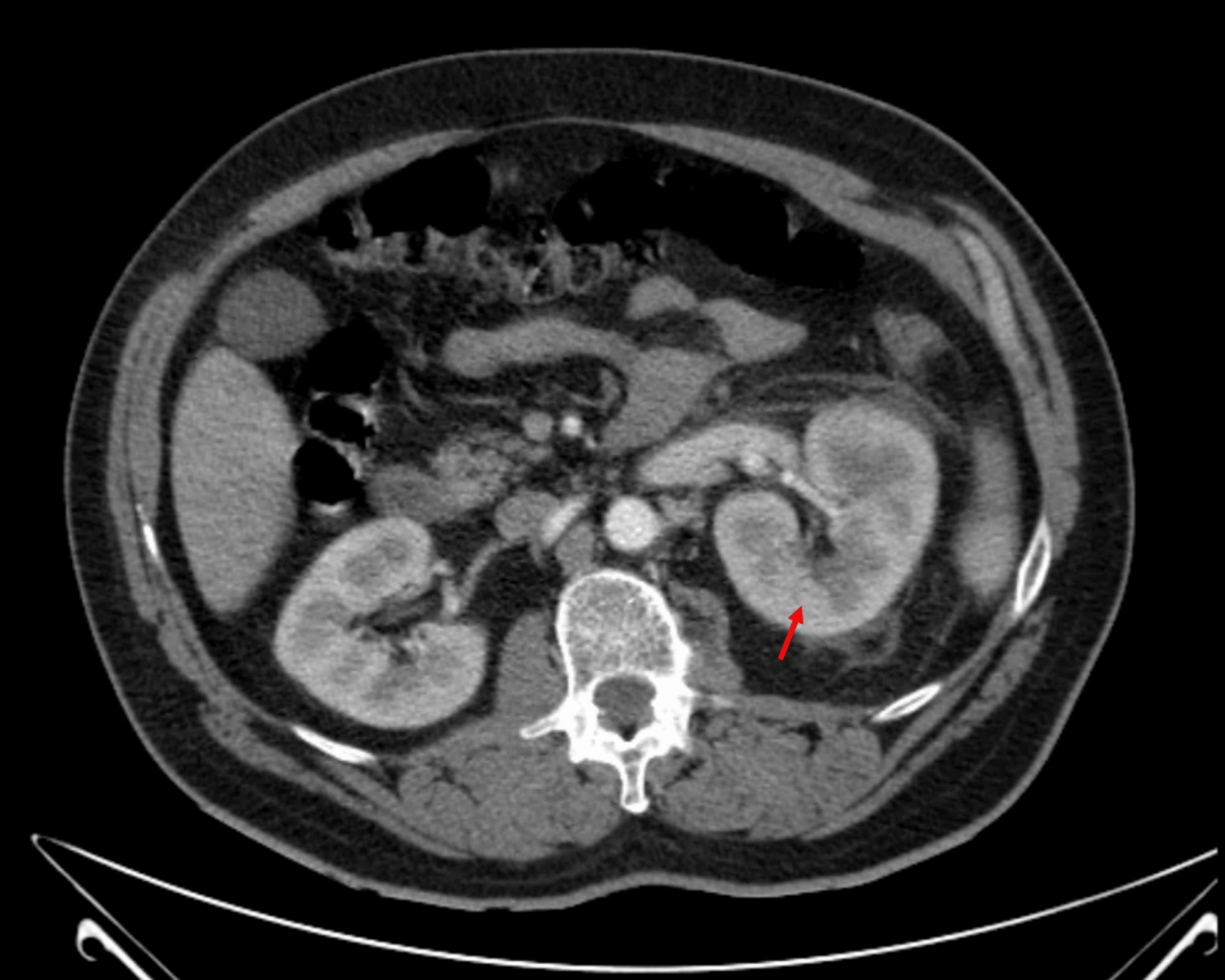
Contrast-enhanced abdominal CT scan shows hypodense area (red arrow), suggestive of focal pyelonephritis and no sign of subcapsular hematoma or other complications.

Laboratory tests showed Hb 14.2 g/dL, leukocytosis (24,200/μL), creatinine 1 mg/dL, C-reactive protein 20.77 mg/dL, and lactate 0.6 mmol/L. Urinalysis revealed leukocytes 10-25/HPF and erythrocytes 5-10/HPF. With the suspicion of acute pyelonephritis, an abdominal CT scan was performed to verify the diagnosis and exclude complications. It showed a hypodense area, with 3.4 cm in the inferior third of the left kidney, suggestive of focal pyelonephritis, without any signs of hematoma, as shown in Figure 1.

**Figure 2:**
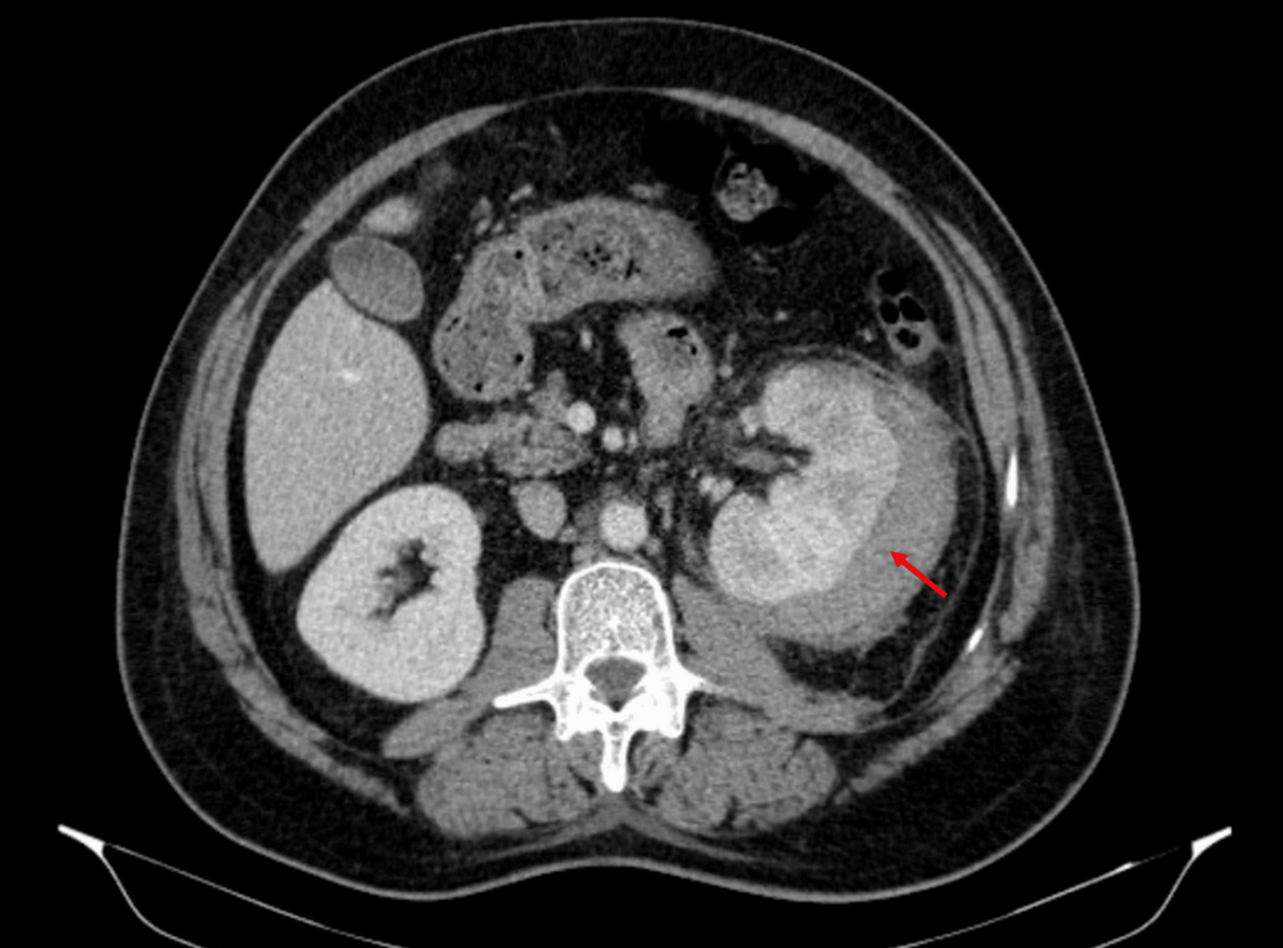
Contrast-enhanced abdominal CT scan shows a left external subcapsular collection, consistent with a subcapsular hematoma (red arrow). No abnormal vascular malformations or tumors were found.

The patient was admitted with the diagnosis of acute pyelonephritis, and blood and urine cultures were obtained. Empirical antibiotic therapy with parental ceftriaxone 2 g once daily was started. During hospitalization, *Klebsiella pneumoniae* was isolated from both blood and urine cultures, and antibiotic therapy was switched to cefotaxime 2 g every eight hours according to the susceptibility testing. Due to persistent fever five days (maximum of 39.4 ºC) after admission despite targeted antibiotic therapy, a transthoracic echocardiogram was performed to rule out endocarditis, showing normal valves without signs of vegetation. Also, an abdominal CT was repeated to rule out a complication, which showed an external subcapsular collection, not seen on the initial CT, measuring approximately 9.1 cm × 7.9 cm × 2.8 cm in its largest longitudinal axes, consistent with a subcapsular hematoma, which can be seen in Figure 2.

The case was discussed with the Urology department, and it was decided to continue with conservative medical management. The patient maintained a fever until the 14th day of hospitalization, so the antibiotic regimen was prolonged. He completed a total of 18 days of parental antibiotic therapy (two days with ceftriaxone, followed by 16 days with cefotaxime). Sequential blood and urine cultures were persistently negative. The patient remained hemodynamically stable despite a drop in hemoglobin from 14.2 to a minimum of 9.9 g/dL.

At the time of discharge, he was apyretic for more than 48 hours. He continued follow-up with the Internal Medicine and Urology department, with a repeat CT scan at one month showing a reduction in hematoma thickness from 2.5 cm to 0.76 cm. At six months, the hematoma had completely resolved.

## Discussion

Subcapsular hematoma of the kidney is an uncommon but significant clinical entity that can pose diagnostic and therapeutic challenges. Typically, subcapsular hematomas are associated with trauma or iatrogenic injury, such as surgical or interventional procedures. Spontaneous subcapsular hematomas are considerably rare and can be attributed to various etiologies [4,5]. These include both benign and malignant neoplasms, which are the most common causes, as well as vascular diseases like polyarteritis nodosa, aneurysms, arteriovenous malformations, portal hypertension, Wegener’s granulomatosis, and renal infarctions. Infection, particularly pyelonephritis, as a cause of spontaneous subcapsular hematoma, is exceedingly rare [4,5].

The clinical presentation of these patients may vary depending on the degree and duration of renal bleeding. Most of the cases reported in the literature present acute abdominal pain, flank mass, and signs of hypovolemic shock, also known as the "Lenk's Triad" [6,7]. 

The management of subcapsular hematoma depends on the patient’s hemodynamic stability and the presence of active bleeding. Treatment options range from conservative medical therapy to embolization of the renal artery or emergency nephrectomy. Conservative treatment involves hydration, pain management, and blood replacement when necessary, and if infection is a concern, antibiotics should be administered [8,9].

The presented case involves a 54-year-old male without high-risk factors, namely diabetes mellitus, anatomic or congenital abnormalities, or immunosuppression, who developed a spontaneous subcapsular hematoma in the context of acute pyelonephritis caused by *Klebsiella pneumoniae* under constant monitoring during hospitalization. This is particularly notable because there was no preceding trauma or known neoplasm, highlighting the need for a high index of suspicion in similar clinical scenarios. Unlike most cases reported [6,7], the patient did not develop "Lenk's triad." The persistence of fever despite targeted antibiotic therapy was the primary symptom that prompted further investigation.

This temporal development of the hematoma underscores the dynamic nature of the condition and the importance of repeat imaging in patients who do not respond as expected to treatment. The absence of abnormal vascular malformations or tumors on imaging further supports the infectious etiology in this case.

Because most of the cases are identified in the context of hypovolemic shock with hemodynamic instability, the treatment is usually more aggressive with the need for percutaneous drainage, embolization of the renal artery, or even emergency nephrectomy [6,7]. In our case, the patient’s prolonged fever resulted in an extended course of antibiotics, ultimately leading to the infection's resolution and a gradual reduction in the hematoma size. Follow-up imaging at one and six months demonstrated significant reduction and eventual resolution of the hematoma, confirming the efficacy of conservative management in this scenario. We could only find one previous report where the patient improved only with conservative medical therapy, demonstrating the rarity of the case reported in this article [3].

## Conclusions

This case highlights the rare but serious complication of spontaneous subcapsular renal hematoma secondary to acute pyelonephritis. It emphasizes the importance of considering this diagnosis in patients with persistent symptoms despite appropriate antibiotic treatment. Clinicians should maintain a broad differential diagnosis and utilize repeat imaging to identify complications that may not be apparent initially. Management should be tailored to the individual patient, with conservative treatment appropriate for hemodynamically stable patients.
